# A systematic review of enhancing urban disaster resilience for smart cities: from a perspective of social network analysis

**DOI:** 10.3389/ijph.2026.1609235

**Published:** 2026-05-08

**Authors:** Yongzhu Zhang, Shiyao Zhu

**Affiliations:** School of Transportation and Civil Engineering, Nantong University, Nantong, China

**Keywords:** multi-agent network, network dynamics, smart city, social network analysis, urban resilience

## Abstract

**Objectives:**

This study examines how Social Network Analysis (SNA) contributes to understanding and enhancing urban resilience, addressing the need to integrate social dimensions beyond technology-driven smart city approaches.

**Methods:**

A systematic literature review combining bibliometric and content analysis was conducted on 89 peer-reviewed articles to identify key themes, node types, and applications of SNA in resilience research.

**Results:**

Findings show that urban resilience is shaped by interactions among community, institutional, and infrastructure networks, though most studies focus on isolated network types. SNA applications have evolved from small-scale surveys to social media and multi-source data, yet longitudinal analyses remain limited. Highly central nodes are often assumed to enhance resilience, but causal relationships are rarely tested. Additionally, social ties, community engagement, and behavioral factors emerge as critical drivers, while challenges persist in data integration, dynamic analysis, and incorporating human behavior into network models.

**Conclusion:**

Advancing SNA for urban resilience requires integrating diverse data sources, longitudinal approaches, and behavioral insights to support more inclusive, adaptive, and evidence-based urban policy and disaster management strategies.

## Introduction

As natural disasters become more frequent and severe, driven by accelerating environmental change, building sustainable urban resilience has emerged as a global imperative [1]. While smart cities are increasingly recognized as key drivers of enhancing urban resilience, their focus has largely centered on technological solutions [2]. Policymakers, urban planners, and researchers alike have embraced the concept of smart cities, wherein advanced technologies such as the Internet of Things (IoT), big data analytics, and remote sensing systems aim to enhance urban functions and disaster preparedness [3]. However, the rapid growth of urban populations and the pace of urbanization have led to new vulnerabilities [4], leaving cities exposed to risks ranging from climate change to pandemics.

Currently, there are approximately 1,000 smart city projects worldwide, spanning Europe, Asia, and North America [5]. These initiatives aim to enhance disaster resilience, but they are not without their limitations. The 2021 flooding disaster in Zhengzhou, China, exemplified these challenges. Despite the city’s smart initiatives, such as intelligent traffic management and environmental monitoring, catastrophic flooding resulted in extensive damage and loss of life, with key vulnerabilities exposed in emergency response systems [6]. Similarly, Typhoon Hagibis in Tokyo in 2019 revealed gaps in disaster preparedness, even in cities with advanced infrastructure [7]. These events underscore the need for a more integrated approach that considers not only technological innovation but also the social systems that underpin urban resilience.

In times of crisis, social networks play a crucial role in information dissemination, resource flow, and community coordination. Effective disaster response cannot rely solely on technology; it must be supported by robust social networks that facilitate communication and collective action. For instance, smart cities cannot ensure public emergency shelters and uninterrupted communications during disasters without robust infrastructure networks [8]. Similarly, safe and efficient transportation during disaster is unattainable without well-developed transportation networks [9]. Furthermore, community networks are essential for incorporating citizens’ opinions and promoting their participation in decision-making processes [10, 11]. Therefore, to improve urban resilience, it is necessary to have a comprehensive understanding of social networks and their role in disaster resistance [12]. Social network is one of the core field considered for smart cities, and social network analysis (SNA) can be employed to examine the relationships between different individuals or organizations [11]. By analyzing the relationship within these networks, insights can be gained into the mechanisms of information transmission and resource distribution [13].

Despite the growing body of literature on SNA and urban resilience, several critical research gaps remain unaddressed. First, existing studies have largely focused on technological innovations in smart cities, with limited integration of social science perspectives, particularly the role of social networks in disaster resilience. Second, while SNA has been applied across various domains, a systematic synthesis of its applications specifically in the context of smart city disaster resilience is lacking. Third, methodological practices across studies have not been critically compared, leaving issues of data integration, longitudinal analysis, and the incorporation of human dynamics largely unexplored. Fourth, the evolution of social networks over time and their impact on urban health and disaster preparedness remain under-synthesized.

Therefore, this systematic literature review bridges the gap between technological innovations in smart cities and the social science perspective, focusing on the role of SNA in enhancing resilience during disasters. By summarizing and analyzing existing research, the paper identifies key progress and gaps, advocating for standardized methods to improve comparability and synthesis. It emphasizes the need for long-term, empirical research to understand how social networks evolve over time and their impact on urban health. The integration of multi-source and longitudinal data, including geospatial and citizen-generated inputs, offers valuable insights for improving disaster preparedness and public health responses. The paper also provides policy recommendations to strengthen social networks, enhancing urban resilience, and outlines future research directions to explore the dynamics of these networks in evolving urban contexts.

## Methods

This paper aims to examine the role of social networks in enhancing the disaster resilience of smart cities, with particular attention to how network-based methods have been applied to understand and support urban sustainability.

### Systematic literature review

To gather relevant literature, three sets of search keywords were utilized: Set 1 for urban resilience (“urban resilience” OR “resilience” OR “resilient city”), Set 2 for smart city (“smart city” OR “digital city”), and Set 3 for social network (“social network analysis” OR “social connections” OR “social network”). Boolean operator AND was applied to combine these three sets. The Web of Science (WoS) core collection database was selected due to its comprehensive coverage across various subjects and its repository of high-quality literature [14]. A single database search may limit the breadth of coverage, given that relevant interdisciplinary studies in smart city technologies and network science also appear in databases such as Scopus, IEEE Xplore, and ScienceDirect. Nevertheless, the WoS was chosen as a rigorous starting point to ensure baseline quality and consistency.

To partially mitigate potential selection bias, a *post hoc* cross validation was conducted by comparing the same key search terms in Scopus and Google Scholar. This cross check confirmed that the core set of highly relevant articles, including all major thematic areas discussed in this review, were consistently indexed across databases, and no critical missing studies were identified.

The language for the literature search has been restricted to English. Specifically, journal articles were targeted, excluding conference papers, working papers, letters, or comments. This decision was based on three methodological considerations. First, journal articles typically undergo a more rigorous and iterative peer review process, which enhances the reliability and validity of reported findings, a critical requirement for methodologically complex approaches such as social network analysis (SNA). Second, journal articles generally provide more complete descriptions of data sources, network construction procedures, and analytical steps, which are essential for reproducing and comparing SNA studies across different urban contexts. Third, the use of journal literature alone offers a more stable and citable baseline for systematic synthesis, as conference proceedings often suffer from variable indexing, inconsistent availability, and lack of version control. The search period was set from 2000 to 2025, resulting in a total of 436 articles meeting the specified criteria.

**FIGURE 1:**
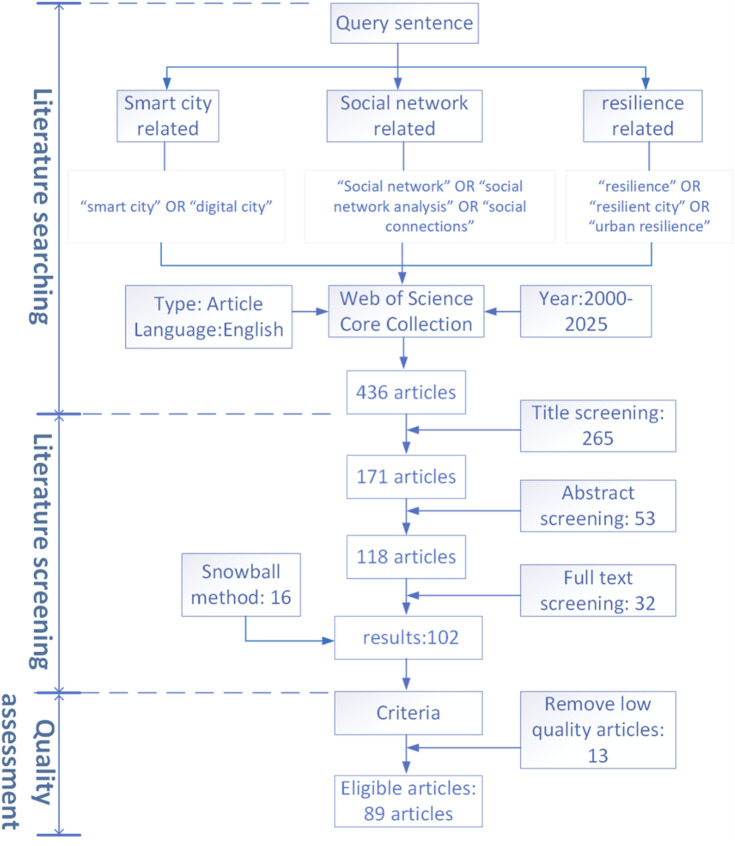
The flowchart of systematic literature review process (Source: Authors own work) (China, 2026).

In order to guarantee the relevance of all results to the topic of this review, a rigorous filtering process was employed, encompassing the assessment of titles, abstracts, and full-text content. Titles and abstracts were screened against predefined inclusion criteria. Specifically, studies were considered eligible if they: (a) employed social network as a core analytical content; (b) focused on urban resilience, disaster resilience, or related concepts; (c) addressed smart city contexts, technologies, or applications; and (d) presented original empirical research or systematic reviews. Subsequently, the full texts of the remaining articles were assessed for eligibility using the same set of criteria. Articles less related to the topic of study, as well as those deemed to have low quality, were excluded. Consequently, 89 articles were selected for a comprehensive final review as shown in Figure 1.

To ensure the methodological rigor and transparency of this systematic review, we developed a structured quality assessment rubric adapted from established frameworks for social network analysis and urban resilience studies. The rubric comprises five criteria: (1) clarity of research objectives, (2) methodological rigor of SNA application, (3) quality and adequacy of data sources, (4) consistency between results and conclusions, and (5) contribution to smart city resilience literature. Each criterion was scored on a 0–2 scale, yielding a maximum total score of 10. Articles scoring below 6 or with any zero score were excluded.

### Content analysis

To deepen the understanding of how social networks contribute to urban resilience in smart cities, this study conducts a comprehensive content analysis based on the systematically reviewed literature. It first explores the studies that adopt SNA as a core methodology for assessing urban resilience, offering insights into how network structures influence smart cities’ capacities to prepare for and respond to disruptions. Then, it explores the temporal and functional evolution of social networks, emphasizing the role of dynamic interactions, feedback loops, and citizen engagement in smart city contexts. Based on the above comprehensive content analysis, the future research directions for social networks are identified and possible suggestions for the enhancement of urban resilience in smart cities are provided.

## Results

### SNA used as a method for smart city resilience

#### Topic of networks

Based on the selected articles, SNA is primarily applied in three key areas: citizen-centered community cooperation and resource sharing, transportation planning, and disaster management. Details are summarized and discussed below.

##### Citizen-centered community cooperation and resource sharing

The selected articles on citizen-centered community cooperation and resource sharing examine various types of networks, such as social networks, social institutional support networks, data sharing and collaboration networks, and social media networks, to understand how communities collaborate, share resources, and enhance resilience. For example, social networks were examined in marginalized rural communities to understand how informal connections among households influence their resilience and access to resources [15]. Similarly, Gomide et al. [16] analyzed social networks within a Southern Amazon Riverside community to identify key individuals and groups that contribute to the flow of resources and support, offering insights for improving primary care delivery [16]. Another study applies SNA to Chinese urban communities, analyzing factors influencing functional loss under flood conditions, using methods like centrality and core/edge structure analysis [17].

Studies also focus on social institutional support networks, such as those in India’s Sudburne region, which help communities adapt to heat stress [18]. Data sharing and collaboration networks, like those in community water quality monitoring projects, explore the impact of centralized versus decentralized structures on decision-making [19]. The paper compares two projects with different network structures, centralized versus decentralized, and examines how these structures influence environmental decision-making at regional and local levels.

Finally, social media networks are examined for their role in information dissemination, particularly in the context of public health. One study analyzes conversations on Twitter to understand the spread of COVID-19 topics and the role of key users in connecting different parts of the network [20]. The other study examines Facebook networks to identify patterns in public health guidance dissemination, using social network analysis to predict useful data patterns and improve public health communication [21].

##### Transportation planning

In transportation planning, SNA focuses on the optimization of transportation and road networks, helping to improve connectivity, efficiency, healthy and safety within transportation systems. Transportation networks, especially road networks, are commonly explored by scholars. Topics like vehicle route prediction [22], critical intersections [23], and traffic planning [24] were discussed. These studies demonstrate that SNA can provide rapid and economical preliminary traffic network analysis, which can effectively guide more detailed traffic planning and operation efforts. Considering the safety of road network, SNA also help to identify and access the organizational resilience, involving information flow patterns and how the network evolves over time, with the goal of improving the implementation of complex road safety interventions [25].

In a different context, carbon emissions are unavoidable in the transportation process, and the network of carbon emissions is also gained interest. Some explored the carbon emission network generated by the cross-district flow of private cars in urban environments [26]. The paper explores how the urban built environment impacts the carbon emissions of private cars, aiming to reveal the spatial correlation of carbon emissions at a micro level.

##### Disaster management

In the context of disaster management, SNA examines networks such as the Humanitarian Action Network, flood management networks, and practical response organization networks. Among them, the Humanitarian Action Network focuses on the relationships between organizations and individuals involved in disaster response [27]. Key stakeholders, such as the government and military, who play significant roles in information sharing were identified. Ways to enhance the participation of other stakeholders and strengthen weaker networks, such as those related to humanitarian aid and resources, were explored. Additionally, a study on social networks among organizations working on heat wave resilience in Los Angeles uses SNA to identify key organizations and assess the cohesiveness of the network [28]. The goal is to enhance climate change adaptation, improve emergency response efforts, and reduce the unequal health impacts of climate change.

Flood management networks are studied to understand the complex relationships and collaboration patterns among social organizations involved in disaster management by extracting data from the Global Knowledge Map [29]. Another study focused on the Actual Response Organization Network, particularly those involved in earthquake emergency relief [30]. It highlights the importance of cooperation between governmental and social organizations, proposing strategies to enhance the adaptability of emergency systems and improve coordination.

SNA also helps explore the role of social media networks in disaster response, illustrating how SNA can be used to coordinate efforts, share information, and mobilize resources effectively during emergencies. Social media networks, particularly those on platforms like Twitter, are analyzed to understand communication patterns during disasters, such as Houstonian Hurricane Harvey [31], flooding in Louisiana, USA [32]. The research focuses on how information is disseminated and how different groups interact, providing insights into the experiences and uncertainties faced by specific groups during disasters.

#### Nodes within SNA

In social network analysis, a node is a basic unit in a network, usually representing an individual, organization, event, or other entity. Nodes are connected to each other through edges to form a network structure [33].

##### Agents

**FIGURE 2:**
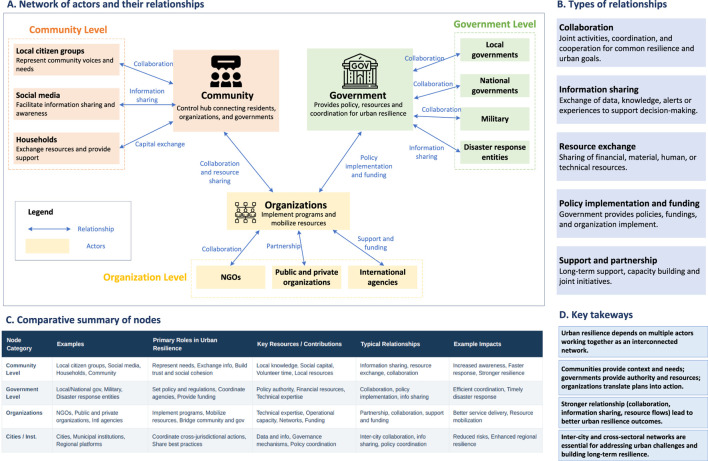
Relationships between different agencies identified from selected papers (Source: Authors own work) (China, 2026).

The common group of nodes in SNA is agents. After summarizing the selected articles, it was found that most of the relationships between different agents involve information exchange or cooperation, as shown in Figure 2.

In community cooperation research, the concept of nodes is defined according to the focus of the study but generally refers to entities or individuals within a network whose interactions have implications for public health. In the context of households, nodes often represent families or individuals exchanging resources such as labor, information, and financial support, which can affect collective health outcomes [15]. Similarly, in health-related studies, nodes may represent individuals or institutions providing formal or informal medical support, with the network connections reflecting exchanges of health information or access to healthcare services, as seen in rural communities where individuals seek medical advice or treatment [18]. In community-based health initiatives, such as water quality monitoring projects, nodes include a range of stakeholders, such as individuals, community groups, and institutions, and their relationships highlight critical factors like data sharing, cooperation, and trust, which directly impact environmental health outcomes [19].

In the broader context of disaster management, nodes are typically the various stakeholders involved in humanitarian networks, including governmental bodies, the military, and aid organizations, all of which play vital roles in public health during crisis situations [20, 21]. These stakeholders collaborate to share critical health information and resources in response to disasters, with government agencies often taking central roles in the flow of information [27]. For example, research on disaster management networks in regions like China’s Greater Bay Area [29] and Los Angeles [28] illustrates how organizations cooperate through social network connections to address health risks related to floods and thermal stress, respectively. These findings emphasize the importance of networked governance and collaboration in enhancing public health preparedness and resilience in the face of environmental challenges.

##### Infrastructures

SNA offers a powerful framework for examining urban infrastructure systems and their influence on public health. In transportation networks, roads, intersections, and traffic lights can be modeled as nodes and edges to analyze mobility efficiency and safety. SNA helps identify critical intersections that affect traffic flow, emergency accessibility, and exposure to air pollution, key determinants of urban health. For instance, studies modeling road networks as graphs [22] and analyzing transportation systems in Louisiana and Mississippi [23] demonstrated that SNA can quickly pinpoint strategic nodes where improved regulation or design could reduce accidents, congestion, and related health risks such as noise and respiratory illnesses.

Beyond mobility, SNA also illuminates the role of social infrastructure, the networks of public spaces, green areas, and community facilities that support physical and mental wellbeing. Research on shrinking cities [34] shows that analyzing these infrastructures as interconnected systems helps identify key spaces that enhance social resilience and population health. Public squares, parks, and health facilities often emerge as central nodes in these networks, fostering social cohesion, promoting active lifestyles, and mitigating the health impacts of urban stressors. Together, these findings position SNA as a crucial tool for designing healthier, more resilient urban environments.

##### Cities or institutions

SNA provides valuable insights into urban public health systems by mapping cities and institutions as nodes and their collaborations as edges. This approach helps reveal how intercity relationships, information flows, and governance structures affect population health outcomes. Cities with high centrality often serve as hubs for coordinating epidemic responses, sharing health innovations, and managing environmental risks. Stronger intercity connections, supported by policy coordination and data sharing, are closely linked to improved regional health resilience and reduced inequalities in health resource distribution.

Empirical evidence highlights how environmental and governance networks shape urban health outcomes [35]. In the Chengdu–Chongqing region, a CO_2_ emission network among 16 cities revealed that tighter intercity cooperation reduced emission intensity and improved air quality, with clear benefits for respiratory health [36]. Similarly, collaborative governance networks addressing air and water pollution in the Beijing–Tianjin–Hebei and Yangtze River Delta regions demonstrate that cross-boundary coordination enhances both ecological sustainability and public health [37]. Together, these studies show that as cities move beyond administrative barriers toward integrated governance, they build more adaptive and health-promoting urban systems.

#### Evaluation indicators for SNA

SNA uses several key indicators to measure network structure and dynamics, each highlighting different aspects of connectivity. Degree centrality counts a node’s direct connections, with high centrality indicating strong connectivity [15]. Betweenness centrality measures a node’s role in connecting pairs of nodes [30], while proximity centrality calculates the sum of shortest paths between a node and all others [27]. Network density is the ratio of actual to possible connections [18], and clustering coefficient indicates the level of node clustering [21]. Other metrics include average degree, path length, and clustering coefficient [29].

Degree, betweenness, and closeness centrality are fundamental metrics. Degree centrality identifies core actors [16], while betweenness centrality highlights nodes controlling resource flow, like critical roadways in flood networks [17]. Closeness centrality measures information diffusion efficiency, as seen in South Korea’s COVID-19 response [33] where high-closeness institutions coordinated actions most rapidly.

Network density and core-periphery structures provide a broader understanding of network morphology and inequality. High density reflects frequent interactions, enhancing community resilience [15]. Core-periphery structures reveal hierarchical differentiation [28], important for policy coordination and disaster management [31]. Cohesive subgroup analysis identifies tightly-knit substructures, supporting regional development strategies.

Eigenvector centrality provides a more sophisticated tool for analyzing complex networks. This metric proves particularly valuable in social media analytics and, as demonstrated in COVID-19 testing networks [21], effectively identifies nodes that are connected to influential neighbors.

#### Methodological heterogeneity in SNA

Based on the summarized empirical studies, a systematic methodological critique reveals significant heterogeneity in how SNA has been operationalized across the reviewed literature.

A fundamental source of variation lies in how researchers define network boundaries. The reviewed studies conceptualize nodes at different levels of analysis: some focus on micro-level actors such as individual households or community members [18], others examine meso-level entities including community organizations and government agencies [25], and still others analyze macro-level units like cities or provinces [35]. Edges are similarly diverse, operationalized as resource exchange [15], information dissemination [20], collaborative behavior [27], or spatial associations derived from gravity models [38].

The reviewed studies employ three main types of data sources, each carrying distinct trade offs that are not always adequately acknowledged. Primary data collected through surveys and interviews capture perceived relationships and informal ties that digital methods cannot observe, but they suffer from small sample sizes and recall bias [16]. Secondary digital data harvested from social media application programming interfaces [17], GPS trajectories [22], or administrative records [39] offer large scale, real time observations, yet they are subject to platform bias, systematically over representing younger, urban, and more affluent populations, and cannot capture offline interactions that often prove critical during disasters. Spatial gravity model based networks enable inter city analysis at regional scales but rely on assumptions about spatial interaction that may not reflect actual collaborative or flow relationships [36]. A significant concern is that each data source captures only a partial view of social networks, yet few studies acknowledge this limitation or attempt multi modal integration.

A third methodological consideration involves how network relationships are mathematically represented. Among the reviewed studies, approximately fifteen studies employed directed networks, primarily those analyzing information flow or resource transfer where directionality matters [31]; the remainder used undirected networks, often treating relationships as inherently symmetric. Regarding edge weights, only nine studies employed weighted networks that capture tie strength through measures such as interaction frequency or collaboration intensity [25]; the majority treated ties as binary, recording only the presence or absence of a connection. This pattern is problematic because disaster response relationships are inherently asymmetric and vary continuously in intensity. A government agency directing NGOs, information flowing from official sources to the public, or resource transfers from donor to recipient cities, none of these are symmetric relationships, yet undirected models treat them as if they were. Similarly, two organizations that communicate daily versus those that communicate only during annual drills are functionally different, yet binary models treat them identically. The reviewed studies utilize a range of SNA software tools. UCINET is the most frequently used, often in combination with NetDraw for visualization. Gephi, R (igraph package) and other tools appear less frequently.

### Dynamics in social networks in smart cities

The dynamics of social networks are a crucial aspect of smart city resilience, with nearly every study in this field addressing the changing nature of these networks [40]. Participants, relationships, social behaviors, and the external environment all experience varying degrees of change, reflecting the network’s dynamic nature [41].

#### Dynamic participants and relationships

One of the most observable dynamics in urban social networks is the continuous change in participants. Urban populations fluctuate due to migration, births, deaths, and demographic shifts, all of which contribute to the evolving composition of social networks. Migration, in particular, plays a significant role in altering the quantity and characteristics of participants, thereby increasing network complexity [42]. Relationships within the network also undergo constant change, influenced by factors such as employment transitions, migration, and the arrival of new organizations [43]. These changes can weaken existing ties and foster new connections, reshaping the social fabric of the city.

#### Influence of social behavior and external environment

Social behavior within smart cities serves as a conduit for communication among participants, influenced by various factors such as emergencies, cultural backgrounds, and technological advancements. For instance, natural disasters tend to intensify interactions among participants, while stable periods see more routine exchanges. The introduction of new technologies, like mobile phones and virtual communication tools, also transforms social behaviors within networks [44]. Additionally, the external environment, including policy frameworks and economic conditions, significantly impacts the dynamics of social networks [45]. Policy frameworks play a pivotal role in shaping a city’s development, guiding urban planners in constructing smart cities that are both functional and aesthetically pleasing. These policies influence the design, infrastructure, and overall trajectory of urban growth, ensuring that smart city initiatives align with broader societal goals and standards [46]. Changes in policy can alter urban social networks by reshaping collaborative relationships, while economic shifts can modify the structure of social networks by introducing new industries and phasing out traditional ones.

#### Methods for analyzing network dynamics

The dynamic nature of social networks can be explored through time series analysis and predictive methods. This approach involves examining historical data to forecast the future developmental trajectory of urban systems [47]. For instance, convolutional neural networks (CNNs) combined with transfer learning have been used for time series flood prediction, providing quick responses to upcoming events, although with varying predictive performance [48]. Another method involves predicting future cumulative precipitation based on past time series data, helping decision-makers assess the need for evacuation orders, though this approach can suffer from information asymmetries during extreme weather events [49]. Additionally, the Long- and Short-term time-series network (LSTNet) method has been applied to forest fire prediction, effectively capturing multi-dimensional relationships between variables but requiring substantial training data to ensure accuracy [50]. However, relying solely on past data for forecasts can be subject to information asymmetries that can lead to less accurate forecasts, especially during extreme weather events. The need for a large amount of training data is another challenge to ensure the effectiveness of the model [51].

### Integrative synthesis: Patterns, trends, and theoretical insights

While the preceding sections catalogued the applications and methodologies of SNA in smart city resilience, this section moves beyond description to synthesize overarching patterns, temporal trends, and theoretical insights emerging from the empirical studies.

#### The multi-network interdependence pattern

A cross-study synthesis reveals that disaster resilience does not depend on a single network but on the interplay among three distinct yet interconnected network types: community networks, institutional networks, and infrastructural or spatial networks. However, most studies focus on only one network type in isolation. Studies of community networks [18] examine local resource exchange and social support but rarely connect these to institutional response structures. Studies of institutional networks [30] map coordination among organizations but seldom examine how community-level ties compensate when formal systems fail. Studies of inter-city spatial networks [37] analyze regional associations using gravity models but cannot capture the ground-level social dynamics that shape actual resilience outcomes.

A critical gap is that cross-network interactions remain severely under-studied. The rare exceptions suggest that community networks become most critical precisely when institutional and infrastructure networks are weakest [32].

#### Temporal trends in SNA methodology

Analyzing the publication years of the reviewed studies reveals a clear methodological evolution over time. Early studies relied mainly on primary data collection through surveys and interviews, with small sample sizes and descriptive network metrics such as density and degree centrality [15]. A noticeable shift occurred around 2015–2019, during which social media data emerged as a dominant source. Studies from this period leveraged Twitter and Facebook APIs to construct large-scale networks (thousands to millions of nodes) and introduced directed and temporal analyses [32]. From 2020 onward, the field has entered an integration phase characterized by three innovations: multi-modal data combining social media with administrative or geospatial sources [17], weighted and directed network models capturing tie strength and directionality [26], and policy-relevant applications linking network structures to real-world governance outcomes [35].

Despite this progress, the field remains overwhelmingly cross-sectional. Only three explicitly analyzed network dynamics over time, and none employed truly longitudinal designs tracking the same network across multiple disaster events. This temporal myopia limits understanding of how networks form, stabilize, or dissolve in response to shocks.

#### The relationship between centrality and resilience

Across multiple studies, a consistent pattern emerges. Nodes with high centrality measures tend to be described as critical for resilience. Government agencies and large NGOs consistently occupy central positions in institutional networks [25], while certain households or individuals serve as information brokers in community networks [18]. In inter-city networks, metropolitan cores such as Shanghai, Nanjing, and Hangzhou exhibit the highest centrality scores [52].

However, a critical observation is that no study has directly tested whether high centrality actually causes better resilience outcomes. Most studies stop at identifying central nodes and asserting their importance. The implicit assumption is that centrality equals influence equals resilience, but this causal chain remains unverified. It is equally plausible that highly central nodes become bottlenecks or single points of failure, or that centrality reflects resource hoarding rather than effective coordination. The field would benefit from hypothesis-driven research that explicitly tests whether, for example, communities with higher network density recover faster from floods, or whether cities with greater betweenness centrality in emission networks achieve steeper emissions reductions.

## Discussion

**FIGURE 3:**
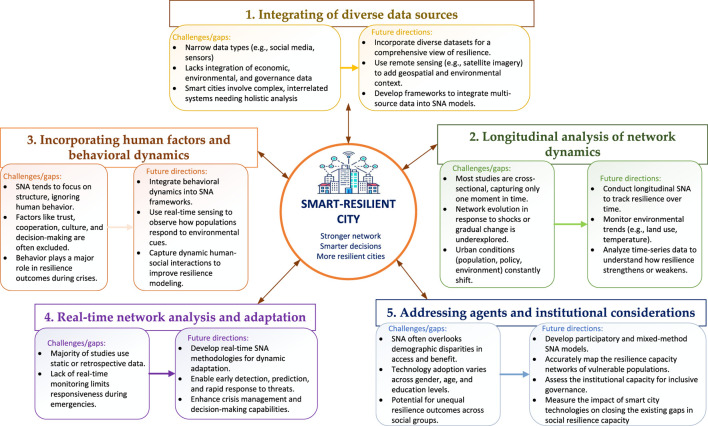
Challenges and future directions (Source: Authors own work) (China, 2026).

Despite the growing body of research exploring the resilience of smart cities through SNA, several gaps remain that warrant further exploration. These gaps present opportunities for advancing the application of SNA in enhancing the resilience of smart cities, particularly in integrating diverse data sources, conducting longitudinal studies, and addressing human and behavioral dynamics. Based on these gaps, future research should focus on several key areas to further advance the role of SNA in enhancing the resilience of smart cities. These future directions aim to address existing challenges and leverage SNA’s potential for improving disaster preparedness, response, and long-term urban sustainability, shown in Figure 3.

### Integrating of diverse data sources

One significant gap in the current literature is the limited integration of diverse data sources within SNA frameworks. While SNA has been effectively used to analyze specific types of networks, such as social interactions or infrastructure connections, studies often rely on a narrow range of data. For instance, many studies focus on social media or sensor data without incorporating critical data related to economics, the environment, or governance, all of which play vital roles in urban resilience. The complexity of smart cities, with their interrelated social, technological, and environmental systems, necessitates a more holistic approach that combines various data types to provide a comprehensive view of resilience [53]. Remote sensing data, such as satellite imagery and aerial photography, can provide real-time, large-scale environmental information [54, 55], enhancing SNA models by adding geospatial context. Future studies could develop methods to incorporate these diverse data sources into SNA, enabling a more thorough understanding of how different factors interact to influence urban resilience.

### Longitudinal analysis of network dynamics in smart city

A significant research gap is the lack of longitudinal studies on how social networks in smart cities evolve, particularly in response to external shocks or gradual changes. Most existing studies offer only a snapshot of network dynamics at a single point in time, limiting our understanding of long-term urban health resilience. As urban environments continuously shift due to factors like population changes, policy adjustments, and economic conditions [45, 46], longitudinal SNA is needed to track how these dynamics influence public health outcomes over time. Remote sensing technologies, which monitor environmental changes such as temperature and vegetation, can complement SNA by providing valuable long-term data to analyze shifts in resilience, offering insights into maintaining urban health amidst evolving challenges [56].

### Incorporating human factors and behavioral dynamics

While SNA excels at analyzing the structural aspects of networks, there is often a lack of focus on human factors and behavioral dynamics, which are critical for understanding resilience. Relationships within social networks are not merely connections between nodes; they are shaped by trust, cooperation, cultural factors, and decision-making processes [57]. These aspects are particularly important in smart cities, where the effectiveness of resilience strategies often depends on how well residents and organizations can work together in times of crisis [58]. Current SNA models adopted in smart cities sometimes overlook these human elements, leading to an incomplete picture of urban resilience. Future research should integrate these human elements into SNA, using real-time environmental data to better understand how behavioral responses interact with social networks and impact public health outcomes.

### Real-time network analysis and adaptation

The dynamic nature of smart cities also calls for the development of real-time SNA methodologies. The ability to monitor and adapt to changing conditions in real time is crucial for enhancing resilience, especially in the face of sudden disruptions such as natural disasters or technological failures [59]. Although the application of real-time SNA in smart cities has made good progress. Most existing studies focus on static or retrospective analyses, which, while valuable, may not provide the immediacy needed for effective crisis management. Advancing real-time SNA techniques could allow cities to better predict and respond to emerging threats, significantly improving their resilience.

### Addressing agents and institutional considerations

A key area for future SNA research in smart city resilience is integrating the heterogeneity of human agents and the role of institutional structures. Current studies often overlook how marginalized groups, local leaders, and diverse demographics are positioned within disaster response networks, potentially leading to unequal health outcomes [60]. For example, perceptions of and access to smart technologies can vary significantly across different social groups, influencing resilience and public health equity [61]. Additionally, the role of institutions such as local governments and NGOs as key network nodes requires further investigation. Future research should focus on participatory SNA models that map the resilience of vulnerable populations, assess institutional capacity for inclusive governance, and measure the impact of smart city technologies on closing social resilience gaps, ensuring equitable and sustainable urban health outcomes.

### Conclusion

The primary goal of this paper is to investigate the role of social networks in enhancing the resilience of smart cities in the face of disasters. By adopting an integrated research approach that combines both quantitative and qualitative methods within a robust review framework, this study meticulously mapped 89 selected articles, analyzing their research type and topics. The literature review identifies key themes central to the paper’s objectives: the use of SNA as a method for enhancing smart city resilience, and the dynamics of social networks within smart city environments. These themes provide a deeper understanding of how social networks operate across various contexts, emphasizing the importance of integrating diverse data sources.

By examining the current body of research, methodologies, and application scenarios, gaps in the existing literature and directions for future studies were identified. Three key findings emerge from the analysis. First, resilience depends on interactions among community, institutional, and spatial networks, yet most studies examine only one network type in isolation. Second, SNA methodologies have evolved from small scale surveys to social media data and then to multi modal integration, but longitudinal designs remain exceptionally rare. Third, nodes with high centrality are consistently described as critical for resilience, yet no study has directly tested this causal assumption.

Future research should focus on integrating multi-source geospatial data, Earth observation data, and citizen-contributed data, conducting longitudinal studies, and addressing the incorporation of behavioral dynamics and equity to provide a more holistic view of urban resilience. The development of real-time SNA tools, as well as the integration of complex network analysis and multi-agent simulations, will enhance the adaptability and effectiveness of resilience strategies in smart cities. These innovations will empower cities to monitor and respond to crises in real time, improving their ability to address both immediate and long-term challenges.

The comprehensive mapping and classification presented in this paper offer valuable insights for scholars, practitioners, and policymakers engaged in smart city resilience research. As research progresses, these insights will inform the design of urban environments that are not only technologically advanced but also capable of ensuring health equity, thereby improving the overall wellbeing of urban populations in the face of future global challenges.
